# Factors influencing the iodine status of children aged 12 to 59 months from Jaffna District, Sri Lanka in the post-iodization era; a descriptive, cross-sectional study

**DOI:** 10.1371/journal.pone.0252548

**Published:** 2021-06-17

**Authors:** Kandeepan Karthigesu, Balakumar Sandrasegarampillai, Vasanthy Arasaratnam

**Affiliations:** Faculty of Medicine, Department of Biochemistry, University of Jaffna, Jaffna, Sri Lanka; University of Agriculture in Krakow, POLAND

## Abstract

**Background:**

Iodine status, including Iodine Deficiency (ID) of the children aged 12–59 months of Jaffna District, Sri Lanka, have never been studied. This study thus aimed to assess ID among children aged 12–59 months by monitoring the Urinary Iodine Concentrations (UIC), the prevalence of goitre, and the factors causing ID.

**Method:**

A cross-sectional study was conducted among 846 children aged 12–59 months in Jaffna District, Sri Lanka. Sociodemographic characteristics and other factors were collected using an interviewer-administered questionnaire. Dietary pattern of children was obtained using semi-quantitative food frequency questionnaire. We performed urinary iodine estimation and physical examinations to detect the goitre, according to the World Health Organization criteria. A multivariate logistic linear regression model was used to identify the factors that causing ID.

**Result:**

The median UIC was 146.4 μg/L (interquartile range = 112.6–185.3 μg/L). Based on the UIC (<100 μg/L), 17.8% had ID, of which 15.7% and 2.1% had mild and moderate ID. The mean consumption of iodine from food was 128.7 (±20.2) μg/day. Gender variation had no influence on ID (p>0.05). Median UIC was significantly associated with living area, wealth status, type of drinking water, and method of iodized salt usage. A higher percentage of ID was significantly associated with younger age [AOR 2.32 (95% CI: 1.31–4.10)], urban area [AOR 1.94 (95% CI 1.27–2.96)], inland regions [AOR 3.20 (95% CI 1.85–5.55)], improper method of iodized salt usage [AOR 3.63 (95% CI: 1.38–9.56)], and low consumption of iodine-containing foods. The neck palpation revealed that only three children had goitre (0.4%).

**Conclusion:**

This study revealed that high ID among the children in Jaffna children was due to improper usage of iodized salt, even though the iodized salt is freely available in the region, living area, and age, while the prevalence of goitre was not significantly identified as a public health problem.

## Introduction

Deficiency of essential micronutrients, including iodine, has substantial impacts on the health and development of growing children [1–3]. Iodine Deficiency (ID) is reported as a public health problem in number of countries [4]. Globally, nearly 2 billion people suffer from ID with around 50 million having clinical manifestations [5]. Children and pregnant women are more vulnerable to develop ID [5] and approximately 266 million school children suffer from ID, with symptoms [6]. ID results in goitre, mental retardation, or reduced cognitive function [5,7–9]. Early screening of iodine status of children is essential to prevent the Iodine Deficiency Disorders (IDDs) [10]. ID is assessed by measuring the Urinary Iodine (UI), which is a useful marker of the recent intake of iodine [11,12]. Iodine level can usually be measured in spot urine specimens as collection of 24-hour urine samples is difficult in a community setting. Relating UI to creatinine is expensive, and it is unnecessary. The median UIC is frequently used than the mean UIC to assess the status of iodine nutrition because the frequency distribution of UI is usually skewed towards elevated values. Thus, ID in a child is defined as a median UIC below 100 μg/L [12].

ID in a geographic area is caused by low iodine content of soil, water or crops [13]. ID, including IDDs, is endemic in the mountainous regions of several countries, including Sri Lanka [5,14–16]. Even though the literacy rate of Sri Lanka is 92.5% [17], the IDDs were high in the Uva Province of Sri Lanka [18]. A recent study in Sri Lanka, excluding the Northern Province, showed that the prevalence of goitre among those who are more than ten years was 8.2%, [19]. Though the government of Sri Lanka commenced the Universal Salt Iodization (USI) programme in 1995 [20], IDDs were known as a major public health problem [14]. Of the iodized salt used by the households of Sri Lanka, 68.3% had an adequate amount of iodine, i.e., >15 ppm [21]. Low iodine content of table salt, consumption of goitrogenic substances containing food (e.g., cassava, cabbage, sweet potato, etc.), living in hilly areas, co-existence of micronutrient deficiencies, deprived socio-economic status of families, low maternal education, age, and sex of the child are associated with the ID [22,23]. Previously, it was reported that the children from Jaffna District were considerably malnourished [24] and were with low food security [25]. A study conducted among the pregnant mothers in Jaffna District revealed that 65.1% of mothers had ID [26].

Iodine status, including prevalence of goitre among the children less than five years and the causative factors of ID have still not been reported from Jaffna District, Sri Lanka. Hence, the children from Jaffna District were selected to find the iodine status, including ID and prevalence of goitre, and the factors contributing to ID were studied.

## Materials and methods

### Materials

All the chemicals used in this research were of analytical grade.

### Study setting

A community-based cross-sectional, descriptive study was conducted between 2012 and 2014 in Jaffna District. Jaffna District is geographically a flat landscape, with a total land area of 1025 km^2^, and a coastline of 160 km [27]. It is surrounded by the Indian Ocean and Jaffna Lagoon, except a narrow connection to the mainland through Elephant-Pass.

### Sample size

The sample size was calculated based on the formula of z^2^p(1-p)/d^2^ to derive the minimum required sample size considering the 95% confidence level, a 5% margin of error (d), a 10% non-response rate, and a design effect of 2. Eventually, a total sample size of 846 was derived.

### Sampling method

A multi-stage cluster random sampling procedure was adopted to select the children proportionate to population size. Medical Officer of Health (MOH) areas of Jaffna District were used as Primary Sampling Units (PSU). From a PSU, required numbers of clusters [Public Health Midwife (PHM) areas] were selected as Secondary Sampling Units (SSU). A total of 10 households were selected randomly from an SSU. If more than a child resides in a house, a child was selected based on his or her recent birthday at the date of data collection.

### Data collection tools and techniques

Age, sex, and birth weight of each child were obtained from the growth chart book, Child Health Development Record (CHDR). The weight of the child was measured with lightweight cloth by using an electronic weighing scale (SECA 811) with an accuracy of ±100g. The height of the child was measured with a portable stadiometer (SECA 213) and Infantometer (SECA 417) with an accuracy of ±0.5 cm according to standard World Health Organization (WHO)’s procedures [28].

Information on the socio-demographic and economic characteristics, drinking water source, and information regarding usage of iodized salt of the selected participants were obtained by a structured interviewer-administered questionnaire. The details of households that fall into the category of urban or rural areas based on the administrative divisions of Jaffna District Secretariat and coastal or inland regions based on the households within the coastal divisions of Grama Niladhari divisions were obtained. A spot morning urine sample of 10mL was collected after first voiding between 08:00 and 12:00 using a sterile wide-mouth plastic urine container with a screw cap covered with black paper. The containers were immediately transferred to a cool box and transported to Biochemistry Research Laboratory, Faculty of Medicine, University of Jaffna for iodine estimation. Collected urine samples were kept at 4°C and analysed within 24 hours.

Training to data collectors (pre-intern doctors) was given by principal investigator and a community physician. The training mainly focused on the study’s objective, the technique of interview, administration of questionnaires, collection, storage, and transport of urine samples, and maintaining ethical issues. The questionnaire prepared in English was translated into the native language, Tamil and then back-translated to English to ensure consistency of translation. A pilot study was carried out to pre-test the questions. During the pre-test, the questionnaire was evaluated for suitability and applicability during the interview between parents and data collectors. All questionnaires were regularly monitored for completeness and consistency by the research supervisors. The overall research activities were coordinated by principal investigator.

### Urinary iodine estimation

The principle of Sandell-Kolthoff reaction was used to determine the iodine concentration in urine samples [12,29]. Briefly, urine was digested with ammonium persulphate at 100°C for an hour. Arsenious acid solution was added into each tube after cooling to room temperature. The tubes were mixed and allowed to stand for 15 minutes. Ceric ammonium sulfate solution was then added to each tube by mixing quickly, at 30-second intervals between successive tubes and allowed to stand at room temperature for 30 minutes. The absorbance was measured at 420 nm [30]. Amount of iodine present in the urine sample was proportional to the disappearance of the yellow colour, which was formed by the reaction between ceric ammonium sulfate and iodine.

### Clinical assessment

Clinical examination of neck for goitre was performed for all children described by Smith et al. (1990) [31]. Goitre classification was done based on the WHO recommended method [12]. Based on that, 0 was graded for no palpable or visible goitre; 1 was graded for palpable, but not visible goitre when the neck is in the normal position; and 2 was graded if swelling in the neck, which is visible when the neck is in a normal position.

### Assessment of iodine intake

To obtain the dietary iodine intake, 24-hour dietary recall method was used with the help of a semi-quantitative Food Frequency Questionnaire (FFQ) as described elsewhere [32–34]. The details on iodine-containing nutrient supplementation, such as Thriposha, Soy flour, multivitamin-multimineral supplements (e.g., Nutromix^TM^) [20], given to children were obtained. The amount of nutrient supplements given to children was recorded.

### Data analysis

Statistical analysis was performed using IBM SPSS Statistics for Windows, Version 27.0 (Armonk, NY: IBM Corp.). Comparisons between means were conducted using an independent Student’s t-test for continuous variables. The chi-square test was used to compare the categorical variables. Significant associated showed in bivariate analysis were entered under multivariate analysis. Non-parametric tests, including the Mann-Whitney U test, Kruskal Wallis multiple comparisons, were performed for median UIC.

### Ethical approval

Ethical approval of the research protocol was obtained from the Ethics Review Committee (ERC) of the Faculty of Medicine, University of Jaffna, Sri Lanka. Informed written consent was obtained from the parents or guardians of the children.

## Results

### Iodine status of children

The median and mean UIC were 146.4 (IQR = 112.6–185.3) and 149.8 (±53.3) μg/L, respectively. It was observed that 17.8% of the children had the UIC<100 μg/L indicating the deficiency of iodine intake, while only 0.8% of the children excreted excessive iodine (UIC>300 μg/L). About 66.0% of the children excreted the normal range of UIC (100–199 μg/L). Only 2.1% of the children had a UIC less than 50 μg/L (moderate ID), while 15.7% of the children had UIC between 50–99 μg/L (mild ID). The children who excreted UIC of more than 200 μg/L was 16.2%. These observations have shown that the children from Jaffna District were not affected with severe ID (UIC<20 μg/L) (Table 1).

**Table 1:** Ranges of urinary Iodine excretion by the children classified based on different criteria such as the socio-demographic factors, birth weight, type of drinking water and usage of iodized salt during cooking.

Variable	Urinary Iodine Excretion (μg/L)					Total No (%)
	20–49 No (%)	50–99 No (%)	100–199 No (%)	200–299 No (%)	>300 No (%)	
***Gender***						
Male	8 (1.9)	67 (16.2)	279 (67.4)	55 (13.3)	5 (1.2)	414 (48.9)
Female	10 (2.3)	66 (15.3)	279 (64.6)	75 (17.4)	2 (0.5)	432 (51.1)
***Age (months)***						
12–23	6 (2.9)	41 (19.6)	117 (56.0)	42 (20.1)	3 (1.4)	209 (24.7)
24–35	3 (1.3)	42 (17.6)	161 (67.4)	31 (13.0)	2 (0.8)	239 (28.3)
36–47	7 (3.2)	30 (13.6)	152 (68.8)	30 (13.6)	2 (0.9)	221 (26.1)
48–59	2 (1.1)	20 (11.3)	128 (72.3)	27 (15.3)	0 (0.0)	177 (20.9)
***Birth weight***						
LBW	4 (3.3)	16 (13.1)	88 (72.1)	14 (11.5)	0 (0.0)	122 (14.4)
NBW	14 (1.9)	117 (16.2)	471 (65.0)	115 (15.9)	7 (1.0)	724 (85.6)
*Living s*ector*						
Urban	8 (3.9)	41 (20.0)	118 (57.6)	36 (17.6)	2 (1.0)	205 (24.2)
Rural	10 (1.6)	92 (14.4)	440 (68.6)	94 (14.7)	5 (0.8)	641 (75.8)
Living area*						
Coastal	0 (0.0)	26 (11.4)	118 (51.8)	80 (35.1)	4 (1.8)	228 (27.0)
Inland	18 (2.9)	107 (17.3)	440 (71.2)	50 (8.1)	3 (0.5)	618 (73.0)
***Type of family***						
Nucleated	11 (2.2)	83 (16.6)	325 (65.0)	78 (15.6)	3 (0.6)	500 (59.1)
Extended	7 (2.0)	50 (14.5)	233 (67.3)	52 (15.0)	4 (1.2)	346 (40.9)
***Family income***						
<14000	0 (0.0)	33 (20.9)	100 (63.3)	25 (15.8)	0 (0.0)	158 (18.7)
14000–16499	4 (2.4)	16 (9.5)	127 (75.1)	22 (13.0)	0 (0.0)	169 (20.0)
16500–20699	5 (2.9)	25 (14.5)	114 (65.9)	28 (16.2)	1 (0.6)	173 (20.4)
20700–29999	3 (2.3)	25 (19.1)	85 (64.9)	17 (13.0)	1 (0.8)	131 (15.5)
>30000	6 (2.8)	34 (15.8)	132 (61.4)	38 (17.7)	5 (2.3)	215 (25.4)
***Wealth status***						
Poor class	0 (0.0)	7 (15.9)	34 (77.3)	3 (6.8)	0 (0.0)	44 (5.2)
Second class	3 (1.4)	31 (14.4)	142 (66.6)	39 (18.1)	0 (0.0)	215 (25.4)
Middle class	15 (3.1)	84 (17.2)	316 (64.8)	69 (14.1)	4 (0.8)	488 (57.7)
Fourth class	0 (0.0)	11 (1.3)	66 (7.8)	19 (2.2)	3 (3.0)	99 (11.7)
***Educational level of mothers***						
No formal education	0 (0.0)	0 (0.0)	4 (100)	0 (0.0)	0 (0.0)	4 (0.5)
Primary	1 (1.3)	8 (12.3)	56 (71.8)	12 (15.4)	1 (1.3)	78 (9.2)
Secondary	16 (2.2)	122 (16.9)	470 (65.1)	108 (15.0)	6 (0.8)	722 (85.3)
Tertiary	1 (2.4)	3 (7.1)	28 (66.7)	10 (23.8)	0 (0.0)	42 (5.0)
***Drinking water***						
Boiled water	9 (1.7)	87 (16.9)	332 (64.3)	83 (16.1)	5 (1.0)	516 (61.0)
Modified water	3 (3.0)	10 (10.1)	68 (68.7)	17 (17.2)	1 (1.0)	99 (11.7)
Unmodified water	6 (2.6)	36 (15.6)	158 (68.4)	30 (13.0)	1 (0.4)	231 (27.3)
Usage of iodized salt**						
Directly added	10 (1.7)	92 (15.2)	398 (65.8)	101 (16.7)	4 (0.7)	605 (71.5)
Added after washing	1 (2.1)	11 (23.4)	30 (63.8)	5 (10.6)	0 (0.0)	47 (5.6)
Solubilized salt	7 (6.8)	21 (20.4)	66 (64.1)	9 (8.7)	0 (0.0)	103 (12.2)
Added after cooking	0 (0.0)	9 (9.9)	64 (70.3)	15 (16.5)	3 (3.3)	91 (10.8)

*Significant level at p≤0.05

** at p≤0.01.

Age was defined in completed months by the date of data collection, and date of birth was obtained from child health development record (CHDR). Low birth weight (LBW) was defined as a child’s birth weight less than 2500 g. NBW-Normal Birth Weight ≥2500 g; LKR-Sri Lankan rupees (1USD = 186 LKR as per 3.1.2020; Boiled water: Water was boiled and cooled; Modified water: Chlorinated or filtered with domestic reverse osmosis plant or traditional water filter; Unmodified water: Groundwater from the open well or tube well without any modification; UIC: 20–49 μg/L- Moderate deficiency; UIC: 50–99 μg/L- Mild deficiency; UIC 100–199 μg/L- Optimal iodine intake; UIC 200–299 μg/L- More than adequate iodine intake; UIC>300 μg/L- Excessive iodine intake.

### Age of the children and iodine deficiency

The age of the children ranged between 12 to 59 months, with the mean of 35 (±13) months. The children were classified based on their age in months and, the median and mean UIC of the male and female children are given in Table 2. When the children of different ages were considered, children having ID (<100 μg/L of UIC) decreased with age, namely those children of 12–23 months, 24–35 months, 36–47 months, and 48–59 months of ages were 22.5%, 18.8%, 16.7%, and 12.4% respectively, and the variations in the percentages were marginally insignificant (p = 0.07). Only 2.9% of the children aged 12–23 months were affected with moderate ID (<50 μg/L), while 1.1% of children aged 48–59 months was affected with moderate ID (<50 μg/L). One-fifth of children (19.6%) aged 12–23 months had mild ID (50–99 μg/L), while 11.3% of children aged 48–59 months had mild ID. There was a significant difference in the iodine excretion related to age (Table 1). Under multiple logistic regression analysis, children aged 12–23 months had a two-fold chance of getting ID than 48–59 months old children [AOR 2.32 (95% CI: 1.31–4.10)] (Table 3).

**Table 2:** Urinary iodine excretion by children classified based on age and sex.

Age (m)*	No. (%)	Total		Males (48.9%; n = 414)						Females (51.1%; n = 432)					
				No. (%)	UIC (μg/L)		Deficient (18.1%; n = 75)			No. (%)	UIC (μg/L)		Deficient (17.6%; n = 76)		
		UIC (μg/L)					No. (%)	UIC (μg/L)					No. (%)	UIC (μg/L)	
		Median	Mean (SD)		Median^#^	Mean (SD) ¤		Median^#^	Mean (SD) ¤		Median^#^	Mean (SD) ¤		Median^#^	Mean (SD) ¤
12–23	209 (24.7)	154.3	154.1 (59.6)	104 (25.1)	152.6^c^	155.5 (60.0)^a^	23 (30.7)	84.5^g^	80.1 (16.1)^e^	105 (24.3)	154.4^cj^	152.7 (59.5)^ai^	24 (31.6)	72.4^g^	71.7 (17.6)^e^
24–35	239 (28.3)	144.3	147.3 (52.8)	123 (29.7)	148.5^c^	152.7 (59.5)^a^	26 (34.7)	67.7^g^	72.3 (18.0)^e^	116 (26.8)	142.7^c^	147.1 (48.8)^a^	19 (25.0)	84.1^fh^	82.5 (12.2)^fk^
36–47	221 (26.1)	140.6	145.2 (51.9)	105 (25.4)	138.2^c^	147.6 (56.5)^a^	14 (18.7)	78.0^g^	72.6 (16.7)^e^	116 (26.8)	138.2^c^	144.6 (55.8)^a^	23 (30.3)	75.6^gh^	71.4 (18.5)^e^
48–59	177 (20.9)	149.1	153.8 (47.2)	82 (19.8)	144.6^c^	146 (47.5)^a^	12 (16)	88.5^g^	78.7 (18.5)^e^	95 (22.0)	159.4^dj^	160.1 (48.8)^bi^	10 (13.2)	77.4^gh^	76.8 (16.4)^ek^
**Total**	**846**	**146.4**	**149.8 (53.3)**	**414**	**146.4**	**148.9 (53.0)**	**75 (18.1)**	**76.0**	**75.8 (17.3)**	**432**	**146.3**	**150.6 (53.6)**	**76 (17.6)**	**77.2**	**75.0 (16.9)**

*Age group in months.

^**#**^Under non-parametric test for medium showed the significant values performed using Mann-Whitney U test.

¤Mean comparison was performed using independent student-t test.

Means within the column without a common superscript letter differ at p≤0.05.

**Table 3:** Selected factors associated with iodine deficiency in children with Odds Ratio (OR) and Adjusted Odds Ratio (AOR). Factors were grouped based on the UIC<100 μg/L.

Independent variable	Iodine deficiency No (%)	OR (95% CI)	AOR (95% CI)
***Gender***			
Male	75 (18.1)	1	
Female	76 (17.6)	1.036 (0.729–1.474)	
***Age (months)***			
12–23	47(22.5)	2.057 (1.184–3.573)	2.319 (1.311–4.102)
26–35	45 (18.8)	1.634 (0.941–2.838)	1.655 (0.939–2.917)
36–47	37 (16.7)	1.424 (0.806–2.207)	1.498 (0.838–2.677
48–59	22 (12.4)	1	1
***Birth weight***			
LBW	20 (16.4)	0.886 (0.529–1.483)	
NBW	131 (18.1)	1	
***Sector***			
Urban	49 (23.9)	1.660 (1.130–2.438)	1.938 (1.269–2.961)
Rural	102 (15.9)	1	1
***Living area***			
Coastal area	26 (11.4)	1	1
Inland area	125 (20.2)	1.969 (1.252–3.099)	3.204 (1.851–5.546)
***Exclusive breastfeeding***			
Yes	93 (17.1)	1	
No	58 (19.3)	1.080 (0.756–1.544)	
Nutrient supplements*			
Yes	140 (17.6)	1	
No	11 (22.0)	1.322 (0.660–2.644)	
***Usage of iodized salt***			
Added after washing	12 (25.5)	3.124 (1.207–8.082)	3.631 (1.379–9.559)
Solubilized salt	28 (27.2)	3.402 (1.508–7.675)	3.743 (1.631–8.587)
Directly added	102 (16.9)	1 (0.899–3.797)	1.962 (0.945–4.076)
Added after cooking	9 (9.9)	1	1
***Drinking water***			
Boiled water	96 (18.6)	1.029 (0.689–1.536)	1.043 (0.687–1.583)
Modified water	13 (11.3)	0.680 (0.347–1.332)	0.725 (0.364–1.446)
Unmodified water	42 (18.2)	1	1

LBW: Low Birth Weight; NBW: Normal Birth Weight.

*Thiriposha, Soy flour, and iodine-containing multivitamin and multimineral supplements.

### Gender and iodine deficiency

There were 414 males (48.9%) and 432 females (51.1%) (Table 2). The median UIC was almost the same in females (146.3 μg/L) and males (146.4 μg/L), while the median value was significantly higher (159.4 μg/L) in females than that in males (144.6 μg/L) aged 48–59 months, whereas median UIC was higher in females than that of males aged 24–35 months (p≤0.05). The median UIC of male (152.6 μg/L) and female (154.4 μg/L) children aged 12–23 months were very close. Overall, the median and mean were significantly not different between the male and female children of 12–59 months. This study demonstrated that 18.1% (75/414) of male and 17.6% (76/432) of female children had ID (p>0.05) (Table 2). Among the iodine deficient children, the least amount of UIC (20–49 μg/L) was observed in 1.9% male and 2.3% female children, while 16.2% of male and 15.3% of female children had UIC between 50–99 μg/L (Table 1).

### Height, weight, and birth weight and iodine deficiency

The mean weight and height of the children were 11.97 (±2.28) kg and 89.87 (±8.69) cm, respectively. The mean height and weight of the males and the female children were 89.71 (±8.75) cm & 12.0 (±2.2) kg, and 90.02 (±8.63) cm & 11.95 (±2.34) kg, respectively (p>0.05). The reference values of the heights and weights of the children of different ages are given in Table 4. The present study showed that the weight of the children was lower than the reference weight of WHO multicentred growth reference study (p≤0.05) [35]. Height and weight of the children of all age groups were not associated with ID (p>0.05) (Table 4).

**Table 4:** Age and sex related to height and weight and iodine status.

Age (m)*	Weight (kg)								Height (cm)							
	Males (n = 414)				Females (n = 432)				Males (n = 414)				Females (n = 432)			
	Iodine Status			RW¤	Iodine Status			RW¤	Iodine Status			RH¤	Iodine Status			RH¤
	D (n = 75) (SD)	MD (n = 8) (SD)	N (n = 339) (SD)		D (n = 76) (SD)	MD (n = 10) (SD)	N (n = 356) (SD)		D (n = 75) (SD)	MD (n = 8) (SD)	N (n = 339) (SD)		D (n = 76) (SD)	MD (n = 10) (SD)	N (n = 356) (SD)	
12–23	10.1 (2.4)	9.5 (2.1)	10.0 (1.8)	10.8	10.5 (2.1)	11.4 (0.9)	9.9 (1.8)	10.1	79.4 (5.5)	78.5 (3.5)	79.2 (5.0)	81.6	80.5 (6.8)	80.6 (3.7)	80.3 (5.5)	80.0
24–35	12.1 (2.0)	10.8 (2.8)	11.8 (1.7)	13.2	11.9 (2.2)	-	11.3 (2.0)	12.6	88.9 (4.9)	82.6 (5.8)	88.0 (5.2)	91.5	88.5 (4.2)	-	87.2 (4.6)	90.2
36–47	12.9 (2.0)	13.2 (1.2)	12.8 (1.6)	15.3	13.0 (1.7)	12.2 (1.8)	12.7 (1.4)	14.9	94.2 (7.0)	95.9 (2.3)	94.4 (4.3)	99.5	95.8 (4.7)	94.3 (4.0)	93.6 (4.7)	98.7
48–59	13.8 (1.7)	12.6	13.7 (1.7)	17.3	12.9 (1.2)	12.4	13.9 (2.3)	17.1	99.3 (3.6)	95	99.4 (4.7)	106.4	97.6 (4.8)	101.3	99.2 (6.1)	105.9

*Age group in months.

D: Deficient children (<100 μg/L); MD: Moderate Deficient children (<50 μg/L); N: An adequate urinary iodine excretion (≥100 μg/L); RW: Reference Weight; RH: Reference Height.

¤WHO-World Health Organization-WHO multi centre growth reference study, 2016 [35].

When the birth weights of the children were considered (Table 1), there has been no association between the birth weight and ID as 16.4% of the low birth weight and 18.1% of the normal birth weight children had ID (UIC <100 μg/L) (p>0.05). Similarly, 3.3% of the low birth weight and 1.9% of the normal birth weight children had moderate ID (UIC <50 μg/L) (p>0.05) (Table 1).

### Demography of living condition and iodine deficiency

#### Living sector: Rural and urban

A high percentage of children were from the rural area (75.8%) was observed (Table 5). Comparatively less ID was observed among the children from rural areas (15.9%) than those from urban areas (23.9%) (p≤0.01) (Table 3). The moderate deficiency was also observed more among the urban children (3.9%) than those from rural areas (1.5%), which was 2.6 times more. The risk of ID among children in urban areas was approximately two times higher than those children in urban areas [AOR 1.94 (95% CI 1.27–2.96)] (Table 3). It was also observed that the children from urban areas had more prevalence of ID than those from rural areas with respect to all age groups. Children aged 12–23 months and 24–35 months from urban areas had a nearly double percentage of ID than children of the same ages from rural areas. Among the rural children, more males (17.7%) were affected than females (14.2%), while among those from urban areas, more females (28.4%) were affected than the males (19.4%). Here too, as far as the ID is concerned, there was no significant difference between the male and female children from urban (p = 0.088) and rural areas (p = 0.139) (Table 5). Though the median UIC was higher in children from rural areas (146.8 μg/L) than those from urban areas (142.6 μg/L), the difference was not significant (p>0.05) (Table 6).

**Table 5:** Age and sex related to the living areas on iodine status.

Age (m*)	Rural (n = 641; 75.8%)								Urban (n = 205; 24.2%)							
	Total	D (15.9%; n = 102) No. (%)	Iodine status of males (n = 311)			Iodine status of females (n = 330)			Total No. (%)	D (23.9%; n = 49) No. (%)	Iodine status males (n = 103)			Iodine status of females (n = 102)		
			D (n = 55) No. (%)	MD (n = 5) No. (%)	N (n = 256) No. (%)	D (n = 47) No. (%)	MD (n = 5) No. (%)	N (n = 283) No. (%)			D (n = 20) No. (%)	MD (n = 3) No. (%)	N (n = 83) No. (%)	D (n = 29) No. (%)	MD (n = 5) No. (%)	N (n = 73) No. (%)
12–23	161	31 (19.3)	15 (27.3)	2 (40.0)	68 (26.6)	16 (34.1)	1 (20.0)	62 (21.9)	16 (33.3)	48	8 (40.0)	0 (0.0)	13 (15.7)	8 (27.6)	3 (60.0)	19 (26.0)
24–35	177	27 (15.3)	16 (29.1)	1 (20.0)	71 (27.7)	11 (23.4)	0 (0.0)	79 (27.9)	18 (29.0)	62	10 (50.0)	2 (66.7)	26 (31.3)	8 (27.6)	0 (0.0)	18 (24.7)
36–47	169	27 (16.0)	13 (23.6)	1 (20.0)	66 (25.8)	14 (29.8)	3 (60.0)	76 (26.8)	10 (19.2)	52	1 (5.0)	1 (33.3)	25 (30.1)	9 (31.0)	2 (40.0)	17 (23.3)
48–59	134	17 (12.7)	11 (20.0)	1 (20.0)	51 (19.9)	6 (12.8)	1 (20)	66 (23.3)	5 (11.6)	43	1 (5.0)	0 (0.0)	19 (22.9)	4 (13.8)	0 (0.0)	19 (26.2

*Age group in months.

D: Iodine deficient children (<100 μg/L); MD: Moderate deficient children (<50 μg/L); N: An adequate urinary iodine excretion (≥100 μg/L).

**Table 6:** Socio-demographic distribution, type of drinking water, and usage of iodized salt with median and mean (SD) urinary iodine concentration.

Variable	No. (%)	UIC (μg/L)		*p*-value for mean UIC	*p-*value for median UIC*
		Median	Mean (SD)		
***Sector***					
Urban	205 (24.2)	142.6	148.4 (59.4)	0.668	0.521
Rural	641 (75.8)	146.8	150.2 (51.3)		
***Living area***					
Coastal	228 (27.0)	152.6	180.2 (59.6)	0.000	0.012
Inland	618 (73.0)	139.4	138.6 (46.1)		
***Type of family***					
Nucleated	500 (59.1)	142.7	148.2 (53.7)	0.303	0.385
Extended	346 (40.9)	149.4	152.1 (52.7)		
***Educational level of mothers***					
No formal education	4 (0.5)	139.7	145.7 (33.8)	0.746	0.818
Primary	78 (9.2)	141.2	151.7 (50.2)		
Secondary	722 (85.5)	146.3	149.1 (54.0)		
Tertiary	42 (5%)	167.0	158.0 (49.1)		
***Total income (LKR/Month)***					
<14000	158 (18.7)	138.5	144.5 (49.7)	0.092	0.320
14000–16499	169 (20.0)	141.0	148.0 (47.6)		
16500–20699	173 (20.4)	146.8	149.9 (56.6)		
20700–29999	131 (15.5)	152.2	144.9 (53.0)		
>30000	215 (25.4)	157.2	158.1 (56.9)		
***Wealth status***					
Poor class	44 (5.2)	140.1^a^	142.1 (40.5)^a^	0.018	0.032
Second class	215 (25.4)	142.6^ab^	149.4 (50.1)^ab^		
Middle class	488 (57.7)	146.3^ac^	147.6 (55.0)^b^		
Fourth class	99 (11.7)	160.8^d^	165.2 (54.8)^ab^		
***Drinking water***					
Boiled water	516 (61.0)	149.2	151.1 (53.6)	0.120	0.052
Modified water	99 (11.7)	152.6	156.2 (55.0)		
Unmodified water	231 (27.3)	139.5	144.2 (51.6)		
***Usage of iodized salt***					
Directly added	605 (71.5)	148.0^a^	152.1 (53.8)^ab^	0.000	0.005
Added after washing	47 (5.6)	138.9^ac^	136.9 (47.7)^bd^		
Solubilized salt	103 (12.2)	132.5^b^	131.0 (48.6)^cd^		
Added after cooking	91 (10.8)	159.0^ad^	162.1 (51.9)^a^		

*Mann-Whitney U test for two independent samples and Kruskal Wallis non-parametric test for median and the significant values had been adjusted by the Bonferroni correction for multiple tests.

Means within the column of the particular variable without a common superscript letter differ at p≤0.05.

#### Living area: Coastal and inland areas

Approximately one-fourth of children (27%) were from coastal areas. It was observed that 75.4% of the children were from rural areas and while 24.6% were from urban areas. A higher median UIC was found in children from coastal areas (152.6 μg/L) than that of children from the inland area (139.4 μg/L) (p≤0.001) (Table 6). About 11.4% of children from coastal areas had ID, while 20.2% of children from inland areas had ID. The risk of ID of children who lived in inland regions was nearly two-fold higher than those children in coastal areas [AOR 3.20 (95% CI 1.85–5.55)] (Table 3).

#### Types of families

More number of children were from nucleated families (59.1%) than from extended families (40.9%). Fewer children from the extended families had ID (16.5%) than those from nucleated families (18.8%). The difference was not statistically significant (p>0.05). Similarly, 23.2% and 21.6% of children aged 12–23 months from the nucleated and extended families, respectively, had the highest ID. The ID among the nucleated and extended families decreased with age. The highest percentage of males from nucleated families had ID (9.8%) than the males in the extended families (7.5%) (p>0.05). However, the females from nucleated and extended families had a similar percentage of ID (Table 7). The median UIC of children from nucleated families (142.7 μg/L) was not statistically different from extended families (149.4 μg/L) (p>0.05) (Table 6).

**Table 7:** Age related to living sector and type of family with iodine deficiency (ID).

Age (m)*	Type of Family											
	Nucleated family (n = 500)						Extended family (n = 346)					
	Total		Males		Females		Total		Males		Females	
	Total	ID (18.8%; n = 94)	Total (n = 247)	ID (n = 49)	Total (n = 253)	ID (n = 45)	Total	ID (16.5%; n = 57)	Total (n = 167)	ID (n = 26)	Total (n = 179)	ID (n = 31)
	No. (%)	No. (%)	No. (%)	No. (%)	No. (%)	No. (%)	No. (%)	No. (%)	No. (%)	No. (%)	No. (%)	No. (%)
12–23	112 (22.4)	26 (23.2)	58 (51.8)	13 (22.4)	54 (48.2)	13 (24.1)	97 (28.0)	21 (21.6)	46 (47.4)	10 (21.7)	51 (52.6)	11 (21.6)
24–35	148 (29.6)	30 (20.3)	76 (51.4)	19 (25.0)	72 (48.6)	11 (15.3)	91 (26.3)	15 (16.4)	47 (51.6)	7 (14.9)	44 (48.4)	8 (18.2)
36–47	127 (25.4)	25 (19.7)	61 (48.0)	9 (14.8)	66 (52.0)	16 (24.2)	94 (27.2)	12 (12.7)	44 (46.8)	5 (11.4)	50 (53.2)	7 (14.0)
48–59	113 (22.6)	13 (11.5)	52 (46.0)	8 (15.4)	61 (54.0)	5 (8.2)	64 (18.5)	9 (14.0)	30 (46.9)	4 (13.3)	34 (53.1)	5 (14.7)

*Age group in months.

ID: Iodine deficiency.

#### Family income

The total family income of a month includes the total income of all the family members. The total family income was classified as pentile based on the Household Income and Expenditure Survey of Sri Lanka, 2016 [36]. When the monthly total family incomes of the families of the children were considered, 18.7% had <LKR 14,000 and 25.4% had >LKR 30,000 (Table 6). Median and mean UIC was highest among the children from families, which had a total family income of >LKR 30,000 (median = 157.2 μg/L and mean = 158.1 μg/L), while the lowest values were observed among the children from the families, which had a total family income of <LKR 14,000 (median = 138.5 μg/L and mean = 144.5 μg/L) (Table 6). ID of children from the family income of <LKR 14,000 had 20.9%, while those from the income of >LKR 30,000 were 18.6%. However, ID of the children was not associated with household income (p>0.05). ID did not associate with age with respect to income categories (Table 8).

**Table 8:** Age related to family income and wealth status of families on iodine deficiency (ID).

Age (m)*	Total Family Income (LKR)									
	<14000 (n = 158)		14000–16499 (n = 169)		16500–20699 (n = 173)		20700–29999 (n = 131)		>30000 (n = 215)	
	Total	ID (20.9%; n = 33)	Total	ID (11.8%; n = 20)	Total	ID (17.3%; n = 30)	Total	ID (21.4%; n = 28)	Total	ID (18.6%; n = 40)
	No. (%)	No. (%)	No. (%)	No. (%)	No. (%)	No. (%)	No. (%)	No. (%)	No. (%)	No. (%)
12–23	37 (23.4)	7 (21.2)	32 (18.9)	7 (35.0)	42 (24.3)	7 23.3)	38 (29.0)	7 (26.9)	60 (27.9)	17 (42.5)
24–35	50 (31.5)	15 (45.4)	43 (25.4)	2 (10.0)	45 (26.0)	6 (20.0)	33 (25.2)	13 (50.0)	68 (31.6)	9 (22.5)
36–47	37 (23.4)	5 (15.2)	44 (26.0)	7 (35.0)	49 (28.3)	10 (33.3)	39 (29.8)	6 (23.1)	5 (24.2)	9 (22.5)
48–59	34 (21.5)	6 (18.2)	50 (29.6)	4 (20.0)	37 (21.4)	7 (23.3)	21 (16.0)	0	35 (16.3)	5 (12.5)
**Wealth Status**										
** **	**Poor class (n = 44)**			**Second class (n = 215)**		**Middle class (n = 488)**			**Fourth class (n = 99)**	
	**Total**		**ID (15.9%; n = 7)**	**Total**	**ID (11.8%; n = 20)**	**Total**	**ID (20.3%; n = 99)**	**Total**		**ID (11.1%; n = 11)**
	**No. (%)**		**No. (%)**	**No. (%)**	**No. (%)**	**No. (%)**	**No. (%)**	**No. (%)**		**No. (%)**
12–23	8 (18.2)		2 (28.6)	51 (23.7)	9 (26.5)	120 (24.6)	33 (33.3)	30 (30.3)		3 (27.3)
24–35	15 (34.1)		2 (28.6)	53 (24.6)	10 (29.4)	143 (29.3)	29 (29.3)	28 (28.3)		4 (36.4)
36–47	8 (18.2)		1 (14.3)	58 (27.0)	7 (20.6)	135 (27.7)	27 (27.3)	20 (20.2)		2 (18.2)
48–59	13 (29.5)		2 (28.6)	53 (24.6)	8 (23.5)	90 (18.4)	10 (10.1)	21 (21.2)		2 (18.2)

*Age group in months.

ID: Iodine deficiency.

#### Wealth status

The wealth status of the families was considered based on the composite measure of the cumulative living standards by assessing household assets, which was assigned a weight or factor score generated through principal components analysis [37]. Among the children, the least numbers were from the poor class (5.2%), followed by the fourth class, i.e., those having rich living standards (11.7%). The majority of the children were from the middle class, 57.7% (Table 6). ID was 15.9% among those from poor wealth status, while 11.1% were from the fourth class (p>0.05). This study clearly showed that among the children from the Jaffna District, the wealth status has no influence on ID. The median UIC was significantly high in children from families of higher wealth status than those from low wealth status. It was found that children from the fourth class excreted higher median UIC (160.8 μg/L) than that of other classes (poor class = 140.1 μg/L) (p≤0.05). ID did not associate with the age of the children considering wealth categories of the families (Table 8).

### Education of the mothers and iodine deficiency

The majority of the mothers (85.5%) had the secondary level of education, whereas 9.2% and 5% had primary and tertiary levels of education, while 0.5% of the mothers did not attend school (Table 6). ID of children was high (11.5%), whose mothers’ educational level was lower (primary education) than that of a higher level of educational level (tertiary education = 9.5%), whereas 19.1% of ID in children was observed among the mothers who has the secondary level of education. The adjusted educational levels of the mothers showed a marginally insignificant difference in ID (p = 0.067).

### Exclusive breastfeeding and nutrient supplementation on iodine deficiency

The present study revealed that 17.1% of children who were EBF had ID than those who did not have EBF (19.3% of children) (p>0.05) (Table 3). Among the children who consumed iodine-containing nutrient supplementation (n = 796), a lower percentage of children (17.6%) had ID compared to children who did not have it (22.0%). Even though nutrient supplementation increased the total iodine consumption of children, it did not significantly improve their iodine status (p>0.05) (Table 3).

### Iodine consumption

#### Drinking water

Twenty-seven percent of children (27.3%) drank water directly from open well/tube well without any modification, while 11.7 and 61% drank the water either chlorinated/filtered and boiled, respectively (Table 6). Among the children using modified water, 8.5% and 3.2% of children used chlorinated water and filtered water, respectively. The ID was low among those who consumed filtered water (7.4%) than among those who used unmodified water (18.2%), boiled (18.6%), and chlorinated water (15.3%) (p>0.05) (Table 1). Even though the ID is affected by different factors, the iodine content of the water in the Dry Zone and coastal area are more than those in the hill area and the Wet Zone [38]. There is no data on the water and soil iodine content for Jaffna District. It is also important to note that the drinking water available in Jaffna District is hard water [39]. ID was decreased with age groups of the children who drank boiled water (Table 9).

**Table 9:** Age related to type of usage of iodized salt and drinking water, on iodine status.

Age (m)*	Usage of iodized salt											
	Directly added			Added after washing			Solubilized salt			Added after cooking		
	(n = 605; 71.5%)			(n = 47; 5.6%)			(n = 103; 12.2%)			(n = 91; 10.8%)		
	Iodine Status			Iodine Status			Iodine Status			Iodine Status		
	D	M	N	D	M	N	D	M	N	D	M	N
	(<100 μg/L)	(<50 μg/L)	No. (%)	(<100 μg/L)	(<50 μg/L)	No. (%)	(<100 μg/L)	(<50 μg/L)	No. (%)	(<100 μg/L)	(<50 μg/L)	No. (%)
	No. (%)	No. (%)		No. (%)	No. (%)		No. (%)	No. (%)		No. (%)	No. (%)	
12–23	35 (34.3)	5 (50)	114 (22.7)	2 (16.7)	1 (100)	7 (20)	9 (32.1)	0	15 (20)	1 (11.1)	0	26 (31.7)
24–35	28 (27.4)	0	134 (26.6)	4 (33.3)	0	10 (28.6)	8 (28.6)	3 (42.9)	26 (34.7)	5 (55.6)	0	24 (29.3)
36–47	25 (24.5)	4 (40)	137 (27.2)	3 (25)	0	10 (28.6)	6 (21.4)	3 (42.9)	18 (24)	3 (33.3)	0	19 (23.2)
48–59	14 (13.7)	1 (10)	118 (23.4)	3 (25)	0	8 (22.8)	5 (17.8)	1 (14.3)	16 (21.3)	0	0	13 (15.8)
**Drinking water**										
	**Boiled water (n = 516; 61.0%)**				**Modified water**^**a**^ **(n = 99; 11.7%)**				**Unmodified water**^**b**^ **(n = 231; 27.3%)**			
	**Iodine Status**				**Iodine Status**				**Iodine Status**			
	**D**	**M**	**N**		**D**	**M**	**N**		**D**	**M**	**N**	
	**(<100 μg/L)**	**(<50 μg/L)**	**No. (%)**		**(<100 μg/L)**	**(<50 μg/L)**	**No. (%)**		**(<100 μg/L)**	**(<50 μg/L)**	**No. (%)**	
	**No. (%)**	**No. (%)**			**No. (%)**	**No. (%)**			**No. (%)**	**No. (%)**		
12–23	34 (35.4)	4 (28.6)	100 (23.8)		2 (15.4)	1 (25.0)	22 (25.6)		11 (26.2)		1 (25.0)	40 (21.2)
24–35	27 (28.1)	6 (42.9)	126 (30.0)		4 (30.8)	1 (25.0)	20 (23.3)		14 (33.3)		0 (0.0)	48 (25.4)
36–47	22 (22.9)	4 (28.6)	109 (26.0)		5 (38.5)	2 (50.0)	23 (26.7)		10 (23.8)		1 (25.0)	52 (27.5)
48–59	13 (13.5)	0 (0.0)	85 (20.2)		2 (15.4)	0 (0.0)	21 (24.4)		7 (16.7)	2 (50.0)	49 (25.9)	

*Age group in months.

^a^Chlorinated or filtered water

^b^Groundwater from the open well or tube well without any modification and it was directly used.

D: Iodine deficient children (<100 μg/L); MD: Moderate deficient children (<50 μg/L); N: An adequate urinary iodine excretion (≥100 μg/L).

#### Usage of iodized salt

It has been observed that all the mothers in Jaffna District used iodized salt as it is freely available in the country. Two commercially available forms of iodized salt, such as crystal and powder forms, are available in Jaffna. In the present study, 71.5% and 10.8% of the mothers directly added the iodized salt before cooking or during the cooking and after cooking, respectively. However, some of the mothers added crystal form of iodized salt after washing (5.6%) and solubilized with water (12.2%) during cooking. Median UIC was significantly higher (159.0 μg/L) in children of mothers who added iodized salt after cooking the foods than those of the mothers who added iodized salt by other methods (Solubilized form of iodized salt = 132.5 μg/L) (Table 6). This study showed the crucial association between ID and the method of iodized salt usage in cooking. The ID was higher in children whose mothers added the iodized salt directly to the food while cooking or before cooking (16.9%); after wash (25.5%); and solubilized with water (27.2%) than that of mothers added after cooking (9.9%). Among the mothers who added iodized salt after cooking, 5.9% of children had excreted a low amount of UI. The children whose mothers added the iodized salt after cooking did not show moderate ID. Children aged 48–59 months whose mothers used iodized salt after cooking excreted an adequate level of iodine. ID was decreased with age groups of children whose mothers used iodized salt either before or during cooking (Table 9). The risk of ID was approximately three-times higher in children when the mothers added the iodized salt after washing with water [AOR 3.63 (95% CI: 1.38–9.56)] or solubilized with water [AOR 3.74 (95% CI: 1.63–8.59)] than that in the children of mothers who added the iodized salt after cooking the foods (Table 3).

#### Dietary habits of the children and iodine from food

Children predominantly consumed cooked rice [85.11 (±24.24) g], food products of rice flour [67.75 (±28.70) g], formula milk [8.58 (±5.21) g], and a variety of legumes daily. They consumed other main foods, including fish [61.8%; 15.58 (±8.05) g], egg [59.7%; 22.79 (±10.57) g], meat [47.9%; 5.01 (±2.17) g], and cow’s milk [38.2%; 234.58 (±120.82) mL] per day. The mean consumption of iodine from food was 128.72 (±20.18) μg/day, which was higher than the World Health Organization’s recommendation [12]. Children obtained a high amount of iodine by consuming milk and rice (37.53 μg/day and 28.09 μg/day, respectively), while nearly 17.58 μg/day was obtained from seafood (Table 10). Children from coastal areas consumed a higher amount of seafood [fish = 26.92 (±7.2) g] than the children from the inland area of the District [fish = 12.24 (±4.42) g] (p≤0.01). Contrary, children from the inland area of the District consumed a higher amount of vegetables than that of children of coastal areas (p≤0.01) (Table 10). This was clearly shown that the mean iodine consumption of coastal children [136.52 (±17.24) μg/day] was significantly higher than the children who live in inland areas of the District [125.84 (±20.41) μg/day] (p≤0.001). Mean consumption of iodine by the vegetarian (1.3%) and the non-vegetarian (98.7%) children were 119.29 (±23.98) μg/day and 128.84 (±20.10) μg/day, respectively. Median UIC was higher among the non-vegetarian children (146.41 μg/L) than that of the vegetarian children (142.32 μg/L) (p>0.05). Though a higher percentage of vegetarian children (18.2%) were affected with ID than the non-vegetarian children (17.8%), it was non-significant (p>0.05). Mean iodine consumption of iodine deficit children [113.12 (±19.36] μg/day) was lower than the non-iodine deficit children [133.30 (±18.11) μg/day] (p≤0.05). It was observed that 16.7% of the children consumed <100 μg/day of iodine from foods based on the available data. Among the children who consumed <100 μg/day of iodine from food, 82.4% of the children had ID.

**Table 10:** Consumption of iodine-rich foods by children from the coastal and inland areas of Jaffna District.

Food item	Iodine Consumption / Day		Total (mean ±SD)	Iodine (μg/day)^a^
	Coastal area	Inland area		
Fish	26.92 (7.21) g	12.24 (4.42)**g	15.57 (8.04) g	15.41
Dry fish	4.72 (1.15) g	3.95 (0.9) g	4.23 (1.08) g	0.97
Shrimp	3.1 (0.75) g	0.61 (0.32)**g	1.05 (0.48) g	0.37
Crab	4.34 (1.43) g	1.85 (0.84)**g	2.45 (1.33) g	0.83
Potato	12.32 (4.02) g	13.57 (5.23) g	12.92 (4.35) g	0.78
Meat	5.4 (2.84) g	6.6 (2.18) g	5.01 (2.17) g	1.70
Egg	14.39 (7.48) g	24.42 (10.52)*g	22.79 (10.57) g	5.24
Milk	232.42 (120.12) mL	235.87 (120.92) mL	234.58 (120.81) mL	37.53
Cooked rice	86.77 (22.63) g	82.41 (24.69) g	85.11 (24.24) g	28.09
Products of rice flour	66.32 (27.25) g	68.33 (25.89) g	67.75 (28.70) g	20.94
Legumes	30.24 (17.29) g	61.91 (21.00)*g	51.35 (20.66) g	Variable
Green leafy vegetable	4.75 (1.31) g	8.42 (3.15)**g	6.22 (3.72) g	Variable
Nutrient supplements	51.59 (29.42) g	56.24 (33.84) g	54.95 (32.47) g	10.77

*P≤0.05 and

**P≤0.01 significant difference.

Food consumption table, Department of Nutrition, Medical Research Institute (MRI), 2007. ^a^USDA, FDA, and ODS-NIH Database for the Iodine Content of Common Foods Release 1.0.2020. Portion size was obtained from mothers by showing the showcards. The portion size was compared with the weight proportion of the portions in grams. Total consumption of foods was summed and divided by number of children who consumed the foods. No data on iodine is available for the food and water sample from Jaffna District.

#### Iodine from nutrient supplementation and foods

Among the children who consumed iodine-containing nutrient supplements (n = 796), the mean consumption of iodine from nutrient supplements was 10.77 (±7.6) μg/day. The mean consumption of total iodine by the study population was 139.5 (±22.12) μg/day (128.72 μg/day from food and 10.77 μg/day from nutrient supplements). The mean iodine consumption from nutrient supplementation was not significantly different between coastal [9.98 (±5.66) μg/day] and inland areas [11.05 (±8.17) μg/day]. The mean total iodine consumption (from foods and supplements) of coastal children [146.43 (±18.33) μg/day] was significantly higher than the children who live in inland areas of the District [136.93 (±22.71) μg/day] (p≤0.0001). Children who had ID consumed mean of 10.46 (±7.6) μg/day iodine from supplements, whereas mean of 10.83 (±7.6) μg/day iodine by non-iodine deficient children (p>0.05). Mean iodine consumption from both food and nutrient supplements of iodine deficit children [123.56 (±20.47) μg/day] was lower than the non-iodine deficit children [142.95 (±20.79) μg/day] (p≤0.001).

### Goitre status of the children

Out of 846 children screened for goitre, only 3 (0.4%) had goitre. On the basis of WHO criteria, among those three children (two females and a male child) who had goitre, two children had grade 1, and a child had grade 2 goitre [40]. However, all the three children excreted the normal level of iodine in their urine with the mean of 185.74 (±51.66) μg/L. It was observed that the mothers of all three children added the iodized salt directly to the food while cooking. Interestingly, two children with goitre were from the inland area of the Jaffna District, whereas a child from the coastal area. Their dietary habits revealed that the mean consumption of fish, shrimp, egg, and milk was 12.21 (±6.42) g, 0.78 (±0.29) g, 18.23 (±9.78) g, and 192.4 (±78.95) mL, respectively. The UIC of a child from coastal areas (215.72 μg/L) was higher than the median UIC of children from inland areas (171.07 μg/L) (p≤0.001). However, mean iodine consumption of all three children [104 (±19.71) μg/day] was significantly lower than the non-goitre children [128.73 (±20.11) μg/day] (p≤0.05). It has been observed that all three children did not consume iodine-containing nutrient supplements.

In contrast, 12%, 5.6%, and 0.6% of children consumed goitrogenic substances containing foods, namely manioc (cassava), cabbage, and sweet potato, respectively. The ID was significantly high (28.3%) in children who consumed goitrogenic substances containing foods than the children who consumed non-goitrogenic substances containing foods (15.6%). Children who consumed goitrogenic substances containing foods had the median UIC of 137.9 μg/L than that of children who consumed non-goitrogenic substances containing foods (151.2 μg/L) (p≤0.05). A child with goitre was from coastal areas and consumed seafood predominantly. Only a child affected with goitre consumed cassava, while the other two children did not consume the goitrogenic foods.

## Discussion

In the present study, the ID, prevalence of goitre and factors influencing ID, including social determinants and dietary patterns in Jaffna children aged 12 to 59 months, were evaluated. This study demonstrated that the median UIC of children of 12 to 59 months was within the normal range (146.4 μg/L), between 100 μg/L and 200 μg/L [12]. However, low iodine excretion (<100 μg/L of UIC) was observed in 17.8% of children. Though the USI programme has been commenced since 1995, IDDs are still the major public health problem in Sri Lanka [18,21]. In a study carried out in Sri Lanka, the mean UIC was 235 μg/L and ranged between 11.1 μg/L and 425 μg/L [41]. It has been reported that the highest median UI level of one of the adjoining provinces of the Northern Province, namely North- Central Province of Sri Lanka, was 231.3 μg/L, and 35.4% of the children had UI levels in the ‘ideal’ range (100–199.9 μg/L), while 30.6% of children had lower UIC (<100 μg/L) [18]. The median value of 146.4 μg/L reported in the present study was significantly lower than the findings of Fernando et al. (2012) [41] and Jayatissa et al. (2005) [18]. The difference could be due to the variation in the age groups and study areas, where Jaffna District occupies the major area of the Jaffna Peninsula. In contrast, the present study on the children from Jaffna District showed that 66% of the children had the UI level within the normal range. Kapil et al. (2014) [42] observed that the median UIC of children of Uttarakhand, India was 125 μg/L. In Jaffna District, maternal median UIC was 140.0 μg/L (IQR = 126.0–268.0 μg/L) [26].

We noticed that 22.5%, 18.8%, 16.7%, and 12.4% of children aged 12–23 months, 24–35 months, 36–47 months, and 48–59 months, respectively, had ID. The increased risk of ID among children of 12–23 months was more when compared with those of 47–59 months. This was supported by a previous study [43]. It was explained that younger children need more iodine than older children [44]. The infant exclusively depends on breastfeeding until six months and then depends on complementary foods, in addition to breast milk [45]. It might be due to an insufficient amount of iodine in early infant feeding [46]. This is supported by our previous study that maternal median UIC (140.0 μg/L) was lower than the WHO recommended reference range for pregnant mothers [12], and 65.1% of the pregnant mothers were reported to have ID (<150 μg/L) [26]. Jayatissa et al. (2013) reported that median UIC in pregnant mothers was substantially lower than the WHO recommendation, and 61.3% of pregnant mothers had ID [47]. The younger children to have ID in the present study could be due to the ID status of the mothers.

On the other hand, the studies among children aged 6–12 years revealed that the older children had a higher risk of getting ID than those of younger ages [22,48]. The older children close to the pubertal growth spurt require a high amount of iodine to support the accelerated growth rate [42,48].

Females aged 6–12 years had higher median UIC than that of male counterparts [48]. We found no difference between the gender in ID and in median UIC. No gender difference in the present study explained the immaturity of androgen and oestrogen hormone in children under 5 years to influence thyroid cell proliferation [49,50].

The weight and height of the children were not significantly affected by iodine-deficient children compared to non-iodine deficient children. Morales-Suárez-Varela et al. (2018) [51] observed that the anthropometry of children did not immediately influence on ID.

The children from rural areas had a higher median UIC and a lower ID than those of children in urban areas. The present study was consistent with previous studies [52,53]. Further, the children from the coastal area had a higher median UIC and lower ID than those from inland areas, which is coherent with previous studies [44,54–56]. Approximately three-quarters of households in the coastal areas also fall under the category of rural areas. This could be due to the consumption of a higher amount of seafood by children in the coastal areas than the children in the inland areas [55,57].

The income and family type of the households were not associated with iodine status. Völzke et al. (2013) [58] reported that the income of the families did not show a statistically significant association with iodine status. In this study, it was also found that the children from a higher wealth status had a higher median UIC and a lower percentage of ID, which is consistent with other studies [59–61]. Parents from a higher wealth household can afford to buy a variety of foods (food diversity) & it determines a higher food security level of households [25,62]. It might also be due to the consumption of processed food outside the home, which contains a higher concentration of iodized salt [48]. It was reported that the median UIC level between groups of wealth index was marginally not associated (p = 0.054) [48].

This study also revealed that utilization of iodized salt to the food preparation by mothers was 100%, which was an ideal value and was higher than the WHO recommendation (>90%) [63]. However, a substantial number of mothers have not practised the recommended method of adding iodized salt while after cooking. We observed that the median UIC level was high in children of mothers who added the iodized salt after cooking. Even though the intake of iodized salt increases the iodine intake, the availability of iodine depends on how the salt is handled [64,65]. In this study population of Jaffna District, 71.5%, 10.8%, 5.6%, and 12.2% of the mothers have added iodized salts directly to the food before or while cooking, after cooking, after washing with water, and after solubilizing with water, respectively. Interestingly, 16.9%, 9.9%, 25.5%, and 27.2% of children had ID, whose mothers added the iodized salt as before or while cooking, after cooking, after washing with water, and after solubilizing with water, respectively. In this study, improper usage of iodized salt during cooking was observed among the majority of the mothers (89.4%). The iodine content and availability of iodine from iodized salt at the household level can vary due to the amount of iodine added during the iodization process; irregular distribution of iodine in the iodized salt within batches and individual bags; losses of iodine during the preparation, transport, and storage in the commercial iodized salt preparation [66]. Further, there are also possibilities for the losses of iodine at household levels due to washing and cooking processes [65–67]. A higher education level of the mothers marginally influenced on the iodine status of children of Jaffna district. A study carried out on children of 5–9 years old in Ratnapura District of Sri Lanka reported that salt crystals were washed before use by 20% of the households. Iodine concentration in salt was significantly lower when the iodized salt is washed before use [64]. It was observed that decreased urinary iodine levels were more in the children whose mothers were using iodized salt in the improper way when compared to the children whose mothers were using the iodized salt after cooking. Thus, even though the iodized salt is available in Jaffna, a significant number of children excreted less amount of iodine with urine. This could be attributed to the improper way of using the iodized salt during cooking and due to the less frequent intake of seafood. It was reported that poor maternal knowledge regarding how to use the iodized salt while cooking was positively associated with iodine status and goitre [68,69].

Even though the mean consumption of iodine from the food and supplementation was higher than the WHO recommendation of ≥90 μg/day [12], 14 children consumed an insufficient amount of iodine per day. However, the nutrient supplementation did not improve the iodine status of the children who consumed it compared to the children who did not consume it. We observed that the mean consumption of iodine-containing nutrient supplements was not satisfactory (mean = 10.77 μg/day). In addition, the iodine level of nutrient supplements, including Soy flour and Thriposha, was approximately between 0.2–0.4 μg/g [70]. Gordon et al. (1998) claimed that iodine supplementation (150 μg iodine/day for 28 weeks) increased the UIC in children who had mild ID (63 μg/L) [71]. Several studies reported that the long duration of iodine supplementation improved the iodine status of children [71,72] and adults [73]. We could not obtain the duration of supplementation. Interestingly, Zimmermann et al. (2000) reported that the effectiveness of iodine supplementation might be affected by iron deficiency anaemia in children [74].

ID in a population is a public health problem if the total goitre rate is more than 5% [12]. Further, when the prevalence of goitre exceeds more than 30% in a population, cretinism may develop up to 5% to 15% of that population [75]. However, in the present study, the prevalence of goitre was 0.4%, indicating that the goitre rate of the children from Jaffna District was not a public health problem [76]. The current prevalence of goitre was lower than the study reports of other parts of the country [18,19,77]. A high prevalence of endemic goitre had been observed in the climatic wet zone of south-west Sri Lanka for several decades than that in the northern dry zone [38], and the water iodine concentration of Jaffna District is still not reported. The high concentration of iodine (up to 84 μg/L) of dry zone water might explain the reason for the low prevalence of goitre in the dry zone area. Fordyce et al. (2000) analysed the iodine level of water in Anuradhapura District [38].

Three children with goitre had excreted the normal level of iodine in their urine [185.74 μg/L]. The children with goitre might have less iodine uptake or excess iodine intake by the thyroid gland or any defect in the thyroid gland in the synthesis of thyroxin hormone; thus, the absorbed iodine is excreted through urine [78]. In the present study, the mean consumption of iodine of all three children (104 μg/day) was higher than the reference intake of iodine per day (≥90 μg/day) [12]. The goitre of the children might also be due to excess iodine consumption. Iodine in the diet is mainly inorganic iodide (I^-^) and is entirely absorbed via the small intestine. However, two-third of the ingested iodine is excreted by the kidney, and the remaining one-third is taken by the thyroid gland [79], where iodine is concentrated by follicular cells and used for thyroxin synthesis [80]. The present study showed the association between goitrogens and UI excretion, whereas the prevalence of goitre was not associated with goitrogen. A similar finding was reported by Fernando et al. (2012) [41] that goitrogens of the foods were not associated with the prevalence of goitre (p≤0.05). Though the goitrogens in some foods are destroyed by cooking, it causes significant inhibition of iodine uptake in the people who coexisted with IDDs and consumed in high amounts. Iodine uptake by the thyroid gland depends on thyroid stimulating hormone, inhibitors such as perchlorate, thiocyanate, and perrhenate in the diet [81] and goitrogens present in some foods.

IDDs prevalence is high in the Jaffna District under the present study settings was related to poor dietary intake of iodine-containing food and importer usage of iodized salt. The present study showed that children who consumed the seafood had less ID. Seafood contain a higher amount of iodine than other foods, including crops [46]. Fish consumption increased the iodine level, while goitrogenic food reduced the iodine status of children [48]. We could not derive a firm conclusion regarding the association between goitre and dietary pattern, vegetarian and non-vegetarian, due to the smaller sample size. The present study has limitations, including the lack of data on the quantity of iodized salt consumed by the children, level of iodine in the salt, and iodine concentration of drinking water. The present study recommends including a larger number of children from vegetarians to derive a better conclusion.

## Conclusions

The present study concluded that the prevalence of ID was high (17.8%). Among the children, 15.7% and 2.1% of children were affected with mild and moderate ID, respectively, while none were affected with severe ID. The mean consumption of iodine from food was (128.7 μg/day) was higher than the World Health Organization’s recommendation. Median UIC was significantly associated with living area, wealth status, type of drinking water, and method of iodized salt usage. Children having ID decreased with age, namely, children aged 12–23 months had a two-fold chance of getting ID than those of 48–59 months. Less ID was observed among the children from rural areas (15.9%) than those from urban areas (23.9%), while children from the inland areas had higher ID (20.2%) than the children from the coastal areas (11.4%). The risk of ID among children in urban areas and inland regions was approximately two- and three-fold higher than those of counterparts, respectively. The increasing educational level of the mothers marginally increased the iodine status of the children. Significantly higher median UIC (159.0 μg/L) was observed in children of mothers who added iodized salt after cooking the foods than those who added iodized salt by other methods. This study also showed that the risk of ID was approximately three times higher in children when the mothers added the iodized salt after washing with water or solubilized with water than that in the children of mothers who added the iodized salt after cooking the foods. The present study also revealed that the consumption of less iodine-rich foods contributed to the ID in Jaffna children. Conversely, ID was not associated with height, weight, and birth weight of the children. Moreover, family income, wealth index, type of drink water, and nutrient supplements were not associated with ID. Consumption of iodine-containing nutrient supplements was insufficient, and it did not improve the iodine status of children. As our study limited to estimate the amount of iodized salt consumed, we recommend incorporating the iodine availability from the table salt. The present study found a low (<5%) prevalence of goitre, and did not have a significant impact on public health.
